# *Weissella cibaria* Attenuated LPS-Induced Dysfunction of Intestinal Epithelial Barrier in a Caco-2 Cell Monolayer Model

**DOI:** 10.3389/fmicb.2020.02039

**Published:** 2020-09-03

**Authors:** Liping Huang, Kang Cui, Wenhao Mao, Yurong Du, Ning Yao, Zhen Li, Huan Zhao, Wang Ma

**Affiliations:** ^1^Department of Oncology, The First Affiliated Hospital of Zhengzhou University, Zhengzhou, China; ^2^Microbiome Laboratory, Henan Provincial People’s Hospital, Zhengzhou, China

**Keywords:** *Weissella cibaria*, tight junction, intestinal barrier, inflammatory response, probiotics

## Abstract

The dysfunction of the intestinal epithelial barrier contributes to local or systemic infection and inflammation. Some lactic acid bacteria (LAB) strains had been shown to improve the conditions of barrier function and, for this reason, are recognized as probiotics. *Weissella cibaria*, a species belonging to the LAB group, is known to promote several health benefits. However, the role of *W. cibaria* in regulating the integrity of the intestinal epithelial barrier has not yet been investigated. In this study, *W. cibaria* MW01 was isolated from Chinese sauerkraut and was selected based on its functional features, such as gastric juice and bile salt tolerance, besides antagonistic activity against pathogenic bacteria. In a cellular model of the intestinal barrier, it was observed that *W. cibaria* was able to adhere more efficiently than *Lactobacillus rhamnosus* GG in Caco-2 cells. Moreover, the LPS-induced inflammation in Caco-2 cells was attenuated by the treatment with *W. cibaria* MW01, which reduced the synthesis of TNF-α, IL-6, and IL-8. In addition, it was noted that the treatment with *W. cibaria* MW01 recovered the integrity of the Caco-2 cell monolayer exposed to LPS. Furthermore, *W. cibaria* MW01 significantly alleviated LPS-induced downregulation of tight junction proteins (TJP) (claudin, occludin, and tight junction protein-1). Mechanistically, *W. cibaria* MW01 inhibited the translocation of NF-κB to the nucleus and deactivated the MLCK-pMLC pathway during LPS exposure. Thus, *W. cibaria* MW01, as a potential probiotic, can protect intestinal epithelial barrier function by regulating inflammation and expression of TJP via the NF-κB-mediated MLCK-pMLC pathway.

## Introduction

The intestinal epithelial barrier, which is composed of the intestinal epithelium, is a tight physical barrier between the mucosal and luminal environments. This barrier serves as a selective permeable barrier for solute, water, and nutrients. In addition, it also constitutes the first line of defense against noxious luminal agents, such as antigens, invasive bacteria, and toxins (Groschwitz and Hogan, 2009; Roehlen et al., 2020). The function of the intestinal barrier is primarily maintained by tight junctions (TJ), which seals the gap between neighboring cells. TJ is a circumferential multiprotein complex located at the apical–lateral regions of intestinal epithelial cells (IECs) and consists of primary transmembrane proteins (e.g., claudins and occludin) and intracellular tight junction proteins (TJP). Claudins and occludin, which can control the structure and permeability of intestinal barrier, are the core of TJ, while TJP plays a structural role in the formation of TJ, connecting the actin cytoskeleton to claudins and occludins (Zihni et al., 2016; Citi, 2018; Zeisel et al., 2018). The expression and localization of these proteins in IECs are dynamically regulated by numerous intracellular and extracellular factors, such as inflammatory cytokines, intestinal microorganisms, and their metabolites (Gonzalez-Mariscal et al., 2003; Chelakkot et al., 2018). TJ dysfunction has been related to an impaired epithelial barrier function, supporting gut leakage that allows bacterial translocation and entry of antigens and toxins to the internal milieu of the body (Citi, 2018). As results, this scenario is favorable to the occurrence and progression of various severe diseases, including inflammatory bowel disease (IBD) (Luissint et al., 2016; Buckley and Turner, 2018), metabolic syndrome (Carratu et al., 1999; Groschwitz and Hogan, 2009), and systemic infection complications (Manfredo Vieira et al., 2018; Khalaf and Sokol, 2020). Thus, regulating TJ proteins to maintain the integrity of the intestinal epithelial barrier may be an effective therapeutic or prophylactic approach to ameliorate the conditions of diseases associated with intestinal barrier disruption (Ulluwishewa et al., 2011; Duan et al., 2019).

Lactic acid bacteria (LAB), as gram-positive bacteria, which are widely distributed in a variety of environments, such as gut microenvironment and traditional fermented food, play a critical role in human life (Mi et al., 2011; Duar et al., 2017). To date, LAB are the most frequently used as probiotics in disease prevention and treatment (Mi et al., 2011). The intervention with probiotic LAB strains, mainly lactobacilli, have been indicated to be a practical approach to improve intestinal epithelial barrier function by directly enhancing the expression and distribution of TJ proteins, attenuating the barrier impairment caused by pathogens toxins, and inflammatory cytokines (Yang et al., 2015; Wang et al., 2016; Zhou et al., 2018). *Weissella cibaria*, belonging to the group of LAB, was first classified in a taxonomic study in 2002 (Bjorkroth et al., 2002). Considering *W. cibaria* a relatively recent member of LAB, the occurrence of potential probiotic strains belonging to this species still needs to be explored. Indeed, some specific *W. cibaria* strains had shown some beneficial effects, demonstrating antioxidant (Ye et al., 2018; Zhu et al., 2018), antimicrobial (Lim et al., 2018), anticancer (Kwak et al., 2018), and immunomodulatory features (Lee et al., 2018; Singh et al., 2018). Singh et al. (2018) indicated that *W. cibaria* 28, a strain isolated from human infant feces, can prevent pro-inflammatory stress in Caco-2 cells challenged with LPS. However, the beneficial effects of *W. cibaria* on the barrier integrity of intestinal epithelial barrier have not been reported.

In this study, three *W. cibaria* strains (MW01, MW02, and MW04) with promising probiotic features were isolated from Chinese sauerkraut, and some functional features, such as tolerance to bile salts and gastric juice, ability to adhere Caco-2 cells, and antagonistic activity against pathogenic bacteria, were assessed. Furthermore, the immunomodulatory effects of these strains were also explored. After these studies, one strain was selected and was subjected to further investigations about their beneficial effects in a cellular model of epithelial barrier disruption triggered by LPS.

## Materials and Methods

### Isolation and Species-Level Identification of LAB Strains

Lactic acid bacteria strains were isolated from Chinese sauerkraut produced by different local household members in Northeast China. For this purpose, the juice of Chinese sauerkraut was serially diluted in sterile saline water. 100 μL of diluent was plated on de Man, Rogosa, and Sharpe agar (MRS agar – Solarbio, Beijing, China) and cultivated at 37°C for 24 h. After incubation, single colonies were randomly isolated and inoculated in MRS broth. 200 μL of bacterial suspension was harvested by centrifugation (6,000 × *g*, 2 min) after being cultivated at 37°C overnight (14–16 h). The harvested cells were washed twice and suspended in sterile saline water. The suspension was boiled at 100°C for 10 min and immediately frozen at −80°C for 10 min. After being thawed at room temperature, the suspension with the released genomic DNA was used as the template in 16S rRNA gene amplification using the universal primers 27F (5′-AGAGTTTGATCCTGGCTCAG-3′) and 1492R (GGTTACCTTGTTACGACTT) (Miller et al., 2013). The sequencing of the 16S rDNA was performed by Shengya Biotechnology Company (Zhengzhou, China). The species-level identification of LAB strains was performed by the Basic Local Alignment Search Tool (BLAST) algorithm from the National Center for Biotechnology Information (NCBI). Stock cultures of LAB strains identified as *W. cibaria* were maintained at −80°C in MRS medium with 20% glycerol.

### Tolerance to Artificial Gastric Juice and Bile Salts

To determine the tolerance of LAB strains to artificial gastric juice and bile salts, which are similar to the conditions of the human upper gastrointestinal tract, an *in vitro* methodology was applied. Briefly, the fresh cultures were centrifuged at 6,000 × *g* for 2 min and washed two times with sterile saline for future use. The bacterial pellets were suspended in artificial gastric juice (MRS broth, pH 2.5, supplemented with 1% (w/v) pepsin) and incubated at 37°C for 4 h. After that, the pellets were harvested and washed two times in sterile saline and resuspended in MRS supplemented with 0.4% (w/v) bile salts (Oxgall, Sigma-Aldrich, Germany). After being incubated at 37°C for 12 h, the bacteria were serially diluted and plated on the MRS agar medium. The bacterial colony counts were calculated to determine the tolerance to artificial gastric juice and bile salts.

### Antagonistic Activity Against Bacterial Pathogens

Antagonistic activity of LAB strains was assessed by the agar well diffusion method. The cultures of LAB strains were centrifuged at 12,000 × *g* at 4°C for 15 min to obtain the cell-free supernatant. To rule out the functions of organic acid, the pH value of the supernatant was adjusted to 7.0 with 2 mol/L NaOH. Moreover, the pH value of MRS broth was adjusted to 4.0 with 2 mol/L lactic acid and was used as control. Next, *Escherichia coli*, *Salmonella enterica*, and *Staphylococcus aureus* were cultivated overnight in LB broth and were spread onto LB agar plates. Then, three Oxford cups were set on the surface of the agar, 200 μl of supernatants was added into each cup, and the plates were incubated at 37°C overnight. The diameter of the inhibition zone around the Oxford cup was measured to determine the antimicrobial activity (Muhammad et al., 2019).

### Adhesion Assay

The human colonic epithelial cell line Caco-2, purchased from ATCC (Rockville, MD, United States), was used to determine the adhesion ability of LAB strains. Caco-2 was cultured in Dulbecco’s Modified Essential Medium (DMEM) supplemented with 10% fetal bovine serum (Thermo Fisher, Waltham, MA, United States) and 1% antibiotics (penicillin–streptomycin solution) (Thermo Fisher, Waltham, MA, United States) at 37°C in 5% CO_2_ incubator. Caco-2 cells were seeded in the transwell insert chambers (1 cm^2^; 0.4 μm pore size, Corning Life Sciences, Tewksbury, MA, United States) at a density of 5 × 10^4^/cm^2^ and grown for 21 days, until the complete differentiation of the cell monolayer. The medium was changed every other day in the first week and every day for the next 2 weeks. Over the cell differentiation period, the *trans*-epithelial electrical resistance (TEER) was assessed with a Millicell ERS-2 Volt-Ohm meter (Merck-Millipore, Burlington, VT, United States).

The adhesion ability assay was performed as previously reported with minor modifications (Reunanen et al., 2015). Caco-2 cells were grown in the transwell insert chambers for 4, 7, 15, and 21 days, respectively. Approximately 10^8^ colony-forming units (CFU) suspended in 1 mL DMEM without antibiotics were added to Caco-2 cell monolayers, which were previously washed twice with phosphate-buffered saline (PBS). After 2 h of incubation, the monolayers were washed three times with PBS to remove the non-adherent bacteria. Then, 1 ml of 1% (v/v) Triton-X was added to lyse the cells and adherent bacteria were serially diluted and plated on MRS agar for calculating the percentage of bacteria adhered to Caco-2 cells. *Lactobacillus rhamnosus* GG was used as a positive control as it has been demonstrated to be an adhesive strain. All experiments were carried out for triplicate analyses.

### Determination of TEER and Inflammation Cytokines

*Trans*-epithelial electrical resistance was measured to assess permeability of the Caco-2 cell monolayer to ionic solutes by using a Millicell ERS-2 Volt-Ohm meter (Merck-Millipore, Burlington, VT, United States). Briefly, the full differentiated cells were maintained in serum-free DMEM for overnight and then treated with LPS (10 μg/ml) for 24 h in the presence or absence of *W. cibaria* MW01, MW02, and MW04 (10^8^ CFU/ml) or treated with strain MW01, MW02, or MW04 alone. The inserts without LPS and bacteria were carried out as a control. Before TEER measurement, the apical and basolateral sides of the Caco-2 cell monolayer were washed and incubated with pre-warmed PBS, and the TEER value measured consecutively for three times. To investigate the immunomodulatory effects of the three *W. cibaria* strains (MW01, MW02, MW04), the cells in the apical sides were collected to determine the gene expression levels of cytokines (TNF-α, IL-6, and IL-8). Furthermore, the medium collected from basolateral sides was used to measure the secretions of these inflammatory cytokines using ELISA kits (BioLegend, San Diego, CA, United States).

### Paracellular Permeability Assay

Paracellular permeability was evaluated in Caco-2 cell monolayers by measuring the flux of fluorescein isothiocyanate–dextran (FITC-dextran – ∼4,000 MWT, Sigma-Aldrich, St. Louis, MO, United States). The medium of fully differentiated Caco-2 cell monolayer was gently removed, and 1 mg/mL FITC-dextran was added to the apical side of the inserts, while 1.5 mL DMEM without serum was added to the basolateral side. After incubation for 6 h, 100 μL basolateral medium was collected and the fluorescence was detected using a microplate reader (Victor X5, PerkinElmer, Waltham, MA, United States) at 480 nm excitation and 520 nm emission wavelengths.

### Quantitative Reverse-Transcription Polymerase Chain Reaction (qRT-PCR)

Total RNA was isolated from Caco-2 cells using TRIzol reagent (Invitrogen, Carlsbad, CA, United States). Next, RNA was reverse-transcribed into cDNA using the PrimeScript RT Reagent Kit with gDNA Eraser (Takara Bio, Kusatsu, Japan). The determination of the mRNA level was performed by quantitative reverse-transcription polymerase chain reaction (qRT-PCR) using iTaq Universal SYBR Green Supermix (Bio-Rad, Hercules, CA, United States). The mRNA levels were normalized to the housekeeping gene GADPH as an endogenous control. The specific gene targets are described in Table 1.

**TABLE 1:** Sequences of primers used for quantitative real-time PCR.

Genes	Primers
TNF-α	F: TCTCGAACCCCGAGTGACAA
	R: TATCTCTCAGCTCCACGCCA
IL-8	F: TGGCTCTCTTGGCAGCCTTC
	R: TGCACCCAGTTTTCCTTGGG
IL-6	F: CATCCTCGACGGCATCTCAG
	R: GCTCTGTTGCCTGGTCCTC
*CLDN1*	F: TGGTCAGGCTCTCTTCACTG
	R: TTGGATAGGGCCTTGGTGTT
*OCLN*	F: GGGCATTGCTCATCCTGAAG
	R: GCCTGTAAGGAGGTGGACTT
*TJP1*	F: TTCACGCAGTTACGAGCAAG
	R: TTGGTGTTTGAAGGCAGAGC
GADPH	F: CTCCTCCTGTTCGACAGTCA
	R: CGACCAAATCCGTTGACTCC

### Western Blotting of TJ Proteins

Caco-2 cells seeded in transwell inserts were incubated for 21 days. Bacteria were added to the inserts, and the insert untreated with bacteria acted as the control group. After 24 h incubation at 37°C, the Caco-2 cells were washed three times with precooled PBS and lysed with 150 μL RIPA lysis buffer (Beyotime Biotechnology, Shanghai, China) for extraction of total proteins. Nuclear proteins in the cell lysate were extracted using the Nuclear Extraction Kit (Cayman Chemical, Ann Arbor, MI, United States). Protein concentration was determined using the BCA protein assay kit (Beyotime Biotechnology, Shanghai, China). The loading buffer was added to the protein samples, and the mixture was boiled for 10 min. The mixture containing 50 g protein was loaded to 10% SDS-PAGE gels to separate the proteins before being transferred to a polyvinylidene difluoride (PVDF) membrane. The membrane was incubated with 5% skim milk at room temperature for 2 h and then incubated with the diluted primary antibodies (1:1000, Invitrogen, Carlsbad, CA, United States) including claudin-1, occludin, tight junction protein-1 (TJP1), NF-κB p65, MLCK, pMLC, MLC, β-actin, or TBP (TATA-binding protein) at 4°C overnight. After being washed twice with Tris buffered saline with Tween 20 (TBST), the membrane was incubated with the secondary antibody anti-rabbit IgG alkaline phosphatase-conjugated produced in goat for 2 h at room temperature. The protein bands were measured and quantified by Gel Doc 116 XR + system (Bio-Rad, Hercules, CA, United States).

### Statistical Analysis

The experiments were conducted in triplicate and repeated three times. The values were expressed as means ± SEM. One-way ANOVA was used to calculate the statistical significance, which was followed by the Dunnett, Turkey’s, or Bonferroni *post hoc* test. *p*-values < 0.05 were considered statistically significant. GraphPad Prism 5.0 software was used for all analyses.

## Results

### Isolation, Species-Level Identification, and Functional Features of LAB Strains Isolated From Chinese Sauerkraut

Two samples of Chinese sauerkraut produced by different local household members were used to isolate the LAB. The sauerkraut samples yielded 40 LAB strains, including 22 lactobacilli strains and 18 *Weissella* strains. Among these *Weissella* strains, 13 were species-level identified as *W. cibaria* and 5 as *W. confuse*. Next, the *W. cibaria* strains were selected to assess their tolerances to artificial gastric juice and bile salts (Table 2). After this assay, four strains were selected based on their best performances against the challenge (MW01, MW02, MW03, and MW04). After 2 h of exposure to artificial gastric juice, strain MW03 seemed to have little effect in gastric juice, with a survival rate of 97.9%. The strains MW01, MW02, and MW04 decreased somewhat, but partially backed up after exposure to bile salts for 12 h, resulting in the final survival rates higher than 86.0%. These results indicated that these four strains may be resistant to stressful gastrointestinal environment. In addition, the antagonistic activity of these strains against *E. coli*, *S. enterica*, and *S. aureus* were also determined. Acidic MRS broth (pH 4.0) showed no inhibition against pathogens. The cell-free supernatants of these *W. cibaria* strains were able to inhibit the growth of these usual enteric pathogens (Table 3). However, neutralized cell-free supernatants lost the antagonistic activity against any of these tested enteric pathogens.

**TABLE 2:** Tolerance to artificial gastric juice and bile salts.

Strains	CFU/ml		
	0 h	4 h, pH 2.5	12 h, 0.4% bile salts
MW01	8.57 ± 0.36 × 10^8^	6.51 ± 0.43 × 10^8^	7.46 ± 0.54 × 10^8^
MW02	8.48 ± 0.22 × 10^8^	6.82 ± 0.57 × 10^8^	7.61 ± 0.25 × 10^8^
MW03	9.22 ± 0.41 × 10^8^	8.98 ± 0.70 × 10^8^	9.03 ± 0.64 × 10^8^
MW04	8.94 ± 0.67 × 10^8^	7.01 ± 0.55 × 10^8^	7.71 ± 0.37 × 10^8^
MW05	8.05 ± 0.14 × 10^8^	5.17 ± 0.03 × 10^6^	5.82 ± 0.34 × 10^6^
MW06	8.76 ± 0.73 × 10^8^	7.20 ± 0.17 × 10^5^	2.79 ± 0.25 × 10^6^
MW07	7.98 ± 0.09 × 10^8^	4.31 ± 1.75 × 10^5^	8.03 ± 0.64 × 10^5^
MW08	8.24 ± 0.07 × 10^8^	7.05 ± 0.25 × 10^6^	5.37 ± 0.37 × 10^7^
MW09	9.32 ± 0.70 × 10^8^	8.12 ± 0.09 × 10^7^	7.03 ± 0.17 × 10^6^
MW10	8.84 ± 1. 26 × 10^8^	8.81 ± 1. 26 × 10^6^	7.84 ± 1. 26 × 10^6^
MW11	8.37 ± 0.06 × 10^8^	9.32 ± 0.06 × 10^5^	4.41 ± 0.06 × 10^6^
MW12	8.93 ± 0.07 × 10^8^	7.51 ± 0.11 × 10^6^	5.97 ± 0.03 × 10^6^
MW13	8.03 ± 0.10 × 10^8^	6.35 ± 0.21 × 10^5^	8.29 ± 0.06 × 10^5^

**TABLE 3:** Antagonistic activity against enteric pathogens.

Strains	Diameter (mm) of inhibition zone		
	*E. coli*	*S. aureus*	*S. enterica*
MW01	24.53 ± 0.83	19.37 ± 0.75	20.64 ± 0.24
MW02	17.06 ± 0.73	20.43 ± 1.08	18.44 ± 0.52
MW03	27.53 ± 0.40	23.53 ± 0.92	20.53 ± 0.88
MW04	16.51 ± 0.68	18.53 ± 0.80	17.50 ± 0.49

### *W. cibaria* Strains Had the Ability to Stably Adhere to Caco-2 Cells

**FIGURE 1:**
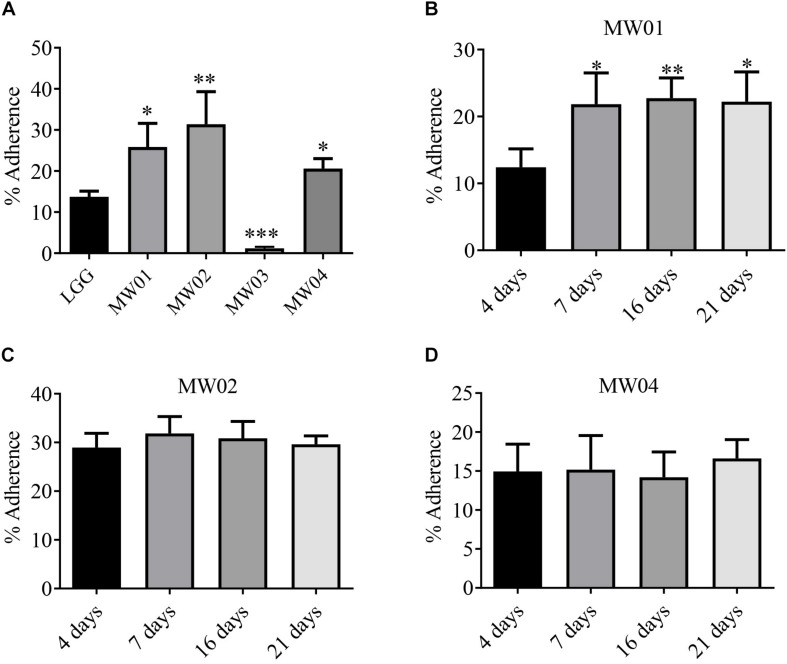
Adhesion assay. **(A)** Adhesive abilities of *W. cibaria* MW01, MW02, MW03, and MW04 to Caco-2 cells were compared to that of *L. rhamnosus* GG (LGG). Adhesive abilities of *W. cibaria* MW01 **(B)**, MW02 **(C)**, and MW04 **(D)** to Caco-2 cells at different growth stages were also evaluated. (*) *p* < 0.05, (**) *p* < 0.005, and (***) *p* < 0.001.

To estimate the adherence capacity of different *W. cibaria* strains to IECs, the differentiated Caco-2 cells were used as an *in vitro* model. As shown in Figure 1A, MW01, MW01, and MW04 strains had a better adhesive capacity than *L. rhamnosus* GG, of which strain MW02 exhibited the best adherence, followed by strains MW01 and MW04. However, MW03 strain, which had more tolerance to artificial gastric juice and bile salts, was unable to effectively adhere to the cells.

In addition, the adherence of MW01, MW02, MW03, and MW04 strains to Caco-2 cells at different time points was studied. In the present study, Caco-2 cells reached confluence after 4 days of growth, and the TEER values of the Caco-2 monolayer were increased continuously from days 5 to 15 and then tended toward stability from 15 to 21 days (Figure S1). Confluent cells (4 days, undifferentiated cells) and cells at three differentiation stages (7, 15, and 21 days) were evaluated. The MW01 strain had adhered better to differentiated Caco-2 cells (Supplementary Figure 1B). The binding abilities of MW02 and MW04 strains were stable over the differentiation period of Caco-2 cells (Figures 1C,D). However, the MW03 strain showed no adhesive properties at different stages of Caco-2 cells (data not shown).

### *W. cibaria* Strains Modulated Immune Responses in Caco-2 Cells Challenged With LPS

**FIGURE 2:**
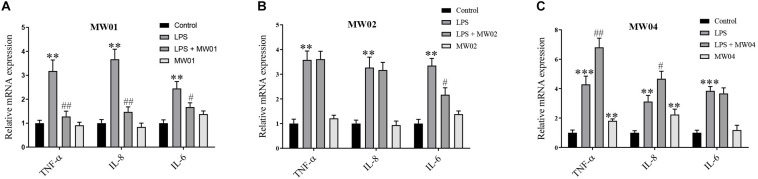
The effect of *W. cibaria* MW01 **(A)**, MW02 **(B)**, and MW04 **(C)** on gene expression of pro-inflammatory cytokines. (**) *p* < 0.005 and (***) *p* < 0.001 versus the control group. (#) *p* < 0.05 and (##) *p* < 0.005 versus LPS group.

It has been reported that pro-inflammatory cytokines can impair the integrity of the intestinal barrier. As shown in Figure 2, Caco-2 cells treated with LPS alone showed a significant increase in transcription levels of TNF-α, IL-6, and IL-8 compared with the control group. However, when Caco-2 cells were treated with LPS simultaneously with the MW01 strain, the mRNA levels of TNF-α, IL-6, and IL-8 were decreased compared with Caco-2 cells challenged with LPS alone. The MW02 strain reduced the mRNA levels of IL-6 in the same scenario stimulated by LPS but had no ameliorative effects on mRNA levels of TNF-α and IL-8. Regarding the treatment with the MW04 strain, an increased expression of TNF-α and IL-8 was observed, indicating that this strain has an immunomodulatory role that directs the expression of pro-inflammatory cytokines. The immunomodulatory effect of *W. cibaria* MW01 was also assessed by ELISA (Supplementary Figure S2). The results confirmed that *W. cibaria* MW01 significantly decreased the secretion of pro-inflammatory cytokines in Caco-2 cells incubated with LPS, which were consistent with the results of mRNA levels observed in Figure 2. Taken together, these results suggested that *W. cibaria* MW01 showed the best anti-inflammatory effects on Caco-2 cells challenged with LPS.

### *W. cibaria* MW01 Improved the Epithelial Barrier of Caco-2 Cells Monolayer Challenged With LPS

**FIGURE 3:**
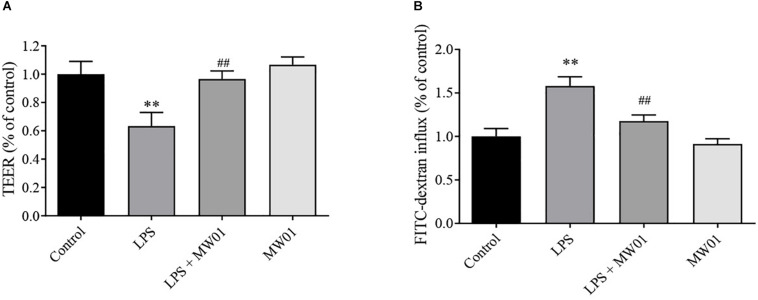
*Weissella cibaria* MW01 improved the epithelial barrier of the Caco-2 cell monolayer challenged with LPS as seen by TEER **(A)** and by FITC-dextran influx **(B)**. (^∗∗^) *p* < 0.005 versus the control group. (##) *p* < 0.005 versus the LPS group.

To evaluate the effect of *W. cibaria* MW01 on the epithelial barrier integrity impaired by LPS, TEER values and FITC-dextran flux were assessed. After 24 h of exposure to LPS, the TEER value of the Caco-2 monolayer was reduced by around 40%. The TEER of the Caco-2 monolayer had no changes when challenged by *W. cibaria* MW01 alone. When the Caco-2 monolayer was challenged by LPS in the presence of *W. cibaria* MW01, the TEER was significantly improved compared with cells that were only challenged by LPS (Figure 3A). In accordance with TEER changes, the increase in FITC-dextran influx caused by LPS was nearly abolished by *W. cibaria* MW01 treatment (Figure 3B). These results indicated that *W. cibaria* MW01 can ameliorate the integrity of epithelial barrier exposed to LPS.

### *W. cibaria* MW01 Changed the Expression Levels of Molecules Related to TJ

**FIGURE 4:**
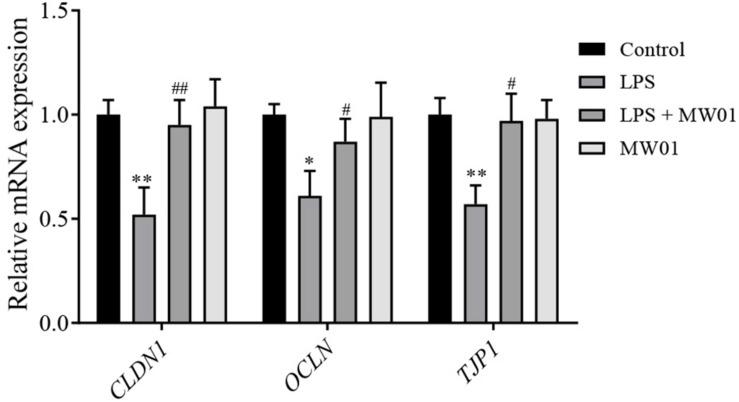
The effect of *W. cibaria* MW01 on mRNA levels of TJ proteins in Caco-2 cells challenged with LPS. (*) *p* < 0.05 and (**) *p* < 0.005 versus control group. (#) *p* < 0.05 and (##) *p* < 0.005 versus LPS group.

The integrity of the intestinal barrier is closely related to the expression levels of TJ proteins, including claudin-1, occludin, and TJP1, which were encoded by CLDN1, OCLN, and TJP1, respectively. To examine whether the MW01 strain affects the mRNA levels of TJ protein genes, the Caco-2 monolayers were challenged by LPS in the presence or absence of the strain. Consistent with previous studies, LPS challenge significantly decreased the mRNA expressions of *CLDN1*, *OCLN*, and *TJP1* versus the control group. In contrast, treatment of challenged Caco-2 cells with *W. cibaria* MW01 improved the mRNA expression of these genes (Figure 4).

**FIGURE 5:**
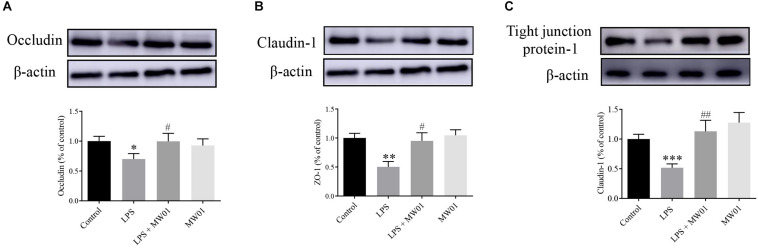
The effect of *W. cibaria* MW01 on protein levels of occludin **(A)**, claudin-1 **(B)**, and tight junction protein-1 **(C)** in Caco-2 cells challenged with LPS. β-Actin was used as the loading control. (*) *p* < 0.05, (**) *p* < 0.005, and (***) *p* < 0.001 versus the control group. (#) *p* < 0.05 and (##) *p* < 0.005 versus the LPS group.

Furthermore, immunoblotting was also performed to investigate the protein levels of claudin-1, occludin, and TJP1. Compared with the control group, LPS decreased the protein levels of claudin-1, occludin, and TJP1. In this same context, the treatment with *W. cibaria* MW01 restored the expression of claudin-1, occludin, and TJP1 in Caco-2 cells challenged with LPS (Figure 5). These results were in agreement with the changes of mRNA levels of these three TJ proteins, indicating that *W. cibaria* MW01 contributes to the integrity of the epithelial barrier through modulating the expression of these TJ proteins.

### *W. cibaria* Improved the Epithelial Barrier Through the Modulation of the NF-K b-Mediated MLCK-pMLC Pathway

**FIGURE 6:**
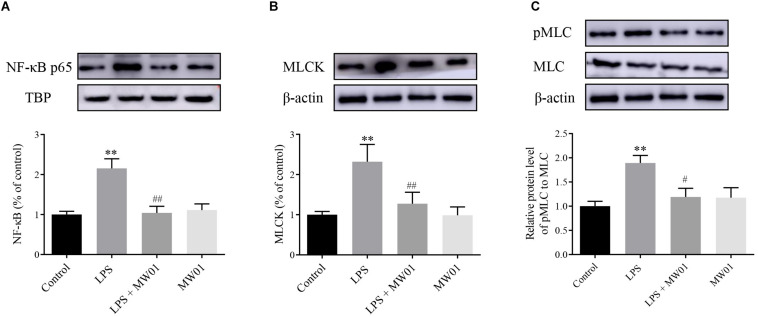
The effect of *W. cibaria* MW01 on protein levels of NF-κB p65 **(A)**, MLCK **(B)**, and pMLC and MLC **(C)** in Caco-2 cells challenged with LPS. TBP was used as the loading control for p65. β-Actin was used as the loading control for MLCK, pMLC, and MLC. (**) *p* < 0.005 versus the control group. (#) *p* < 0.05 and (##) *p* < 0.005 versus LPS group.

MLCK, a serine/threonine protein kinase, modulates the construction of actin–myosin structures and consequent paracellular permeability through promoting MLC phosphorylation. Thus, the expression of MLCK and pMLC can be considered as indicators of the IEC barrier disruption induced by pro-inflammatory cytokines. Meanwhile, NF-κB, a key factor driving the expression of pro-inflammatory cytokines, contributes to the stimulation of the MLCK-pMLC pathway, which can be indicative of the IEC barrier disruption. Therefore, the NF-κB-MLCK-pMLC signaling pathway in Caco-2 cells was analyzed in the presence of *W. cibaria* MW01 and/or LPS. The activation of NF-κB was determined by the accumulation of NF-κB p65 translocated to the nucleus. Western blotting results showed that when incubated with LPS alone for 24 h, the amount of NF-κB p65 in nuclei of Caco-2 cells was increased. Administration of *W. cibaria* MW01 antagonized the nuclear translocation of NF-κB p65 to nuclei in Caco-2 cells challenged by LPS (Figure 6A). LPS also activated the MLCK expression (Figure 6B) and consequently increased the MLC phosphorylation. However, *W. cibaria* MW01 treatment reduced the activation of the MLCK-pMLC signaling pathway induced by LPS (Figure 6C). These results suggested that *W. cibaria* MW01 may play its beneficial effects by modulating the inflammatory responses and the expression of TJ proteins through the NF-κB-mediated MLCK-pMLC signaling pathway.

## Discussion

Lactic acid bacteria, one of the predominant bacterial groups in traditional fermented foods, play an important role in improving the texture, aroma, flavor, and taste of food materials (Tamang et al., 2016). In addition, some strains of LAB isolated from fermented foods also have shown probiotic features. Through the consumption of fermented foods, LAB could provide many health benefits, including improving the gut microenvironment, lowering the risk of diabetes and cardiovascular diseases, and regulating immunity (Rezac et al., 2018). Traditional fermented foods have been used to isolate new potentially probiotics strains. Indeed, *W. cibaria* JW15, isolated from kimchi, has been recognized to enhance immune and natural killer activity in animal and clinical trials (Lee et al., 2018; Yu et al., 2019; Park et al., 2020). The microbiota of the naturally fermented Chinese sauerkraut contains LAB strains and, consequently, could be a source of potential probiotics. In this study, four *W. cibaria* strains were isolated from sauerkraut in Northeast China, which showed tolerance to artificial and bile salts, indicating that they may survive in the harsh conditions of the gastrointestinal environment. It has been reported that gastric acidity and bile salts constitute the main defense of most ingested microorganisms. Moreover, the acidic cell-free supernatants of these four isolates inhibited the growth of the common pathogens, indicating that they are likely to intervene gastrointestinal infections by producing antibacterial active substances, and for this reason, these four strains have potential to be used as probiotics. In addition, the acid MRS broth (pH 4.0) was not able to inhibit the pathogens, and the supernatants lost their inhibition abilities against the tested pathogen after being neutralized. These results indicated that the antibacterial activity of *W. cibaria* strains may be attributed to acid-dependent substance, but not organic acids. It has been well known that LAB can produce various antimicrobial metabolites, such as hydrogen peroxide, bacteriocins, and peptides besides organic acids (Reis et al., 2012). Thus, further studies about the antibacterial compounds produced by these *W. cibaria* strains are still necessary.

The adhesion to intestinal mucosa is considered one of the prerequisites for a probiotic strain to be able to exert its beneficial effects. Adherence may facilitate the colonization of probiotics in the gastrointestinal tract and could ensure the sustainability of health benefits, such as occupation of specific niches impairing the adhesion of pathogenic bacteria (Hibbing et al., 2010), modulation of immune system (Corthe`sy et al., 2007), and protection of IEC barrier integrity (Chelakkot et al., 2018). In this study, *W. cibaria* MW03 showed no binding to Caco-2 cells, neither differentiated monolayer nor undifferentiated Caco-2 cells. The other three *W. cibaria* (MW01, MW02, and MW04) strains were able to adhere to Caco-2 cells with different adhesive efficiencies. *In vivo*, IECs are constantly renewed and differentiated to perform replacement of old IECs by immature IECs (Vereecke et al., 2011). The different profiles of surface molecules expressed in IECs at differentiated stages affect the adherence of bacteria to them (García-Lorenzo et al., 2012). Interestingly, the adhesive ability of the MW01 strain was dependent on the differentiation stage of Caco-2 cells, since it seemed that this strain preferred to adhere to differentiated Caco-2 cells. However, the binding abilities of MW02 and MW04 strains showed no differences among differentiation stages of Caco-2 cells. Our result indicated that the adhesive abilities of these strains are strain-specific. The underlying mechanisms of different adhesive abilities of these four strains need to be further studied.

Besides the absorptive role and formation of the physical barrier, IECs can also play a role in expressions of cytokines and chemokines, which can orchestrate immune responses (Okumura and Takeda, 2017). The intestinal barrier is chronically exposed to various antigens including microorganisms and toxin (Gu et al., 2016). LPS, an endotoxin derived from gut gram-negative bacteria, mediates inflammatory reaction and homeostasis disturbance by stimulating the massive production of pro-inflammatory cytokines, such as IL-6, IL-8, and TNF-α (Singh et al., 2018; Wang and Kong, 2018). It has been reported that probiotics could directly interact with the IECs regulating the immune inflammation caused by LPS (Singh et al., 2018). In this study, the MW01 strain reduced the expression and secretion of IL-6, IL-8, and TNF-α in LPS-challenged Caco-2 cells. In contrast, the other two strains modulated the LPS-mediated inflammatory responses with varying degrees, which is similar with the phenomenon described in previous research (Singh et al., 2018). Moreover, the protective effect of *W. cibaria* MW01 on inflammatory response induced by LPS can play an active role in limiting the inflammatory responses during chronic intestinal inflammation to improve the physiological conditions of intestine.

Many studies have confirmed that intestinal inflammation, caused by pro-inflammatory cytokines, can disrupt IEC barrier integrity (Chen et al., 2016). As the largest and most important barrier integrating external and internal signals to recognize self from non-self, its integrity is the foundation for performing the normal barrier function. Any disruption of the barrier integrity can lead to local or systemic infection and inflammation (Groschwitz and Hogan, 2009; Citi, 2018). In this study, the protective effect of the MW01 strain in IEC integrity was assessed using the Caco-2 cell monolayer challenged by LPS. TEER and FITC-dextran influx were used to evaluate the integrity of *de novo* established endothelial or epithelial monolayers. Consistent with previous reports (Guo et al., 2015), LPS challenge can lead to TEER reduction and increased FITC-dextran influx, which were attenuated by *W. cibaria* MW01. These improvements of epithelial barrier integrity may be due to an increased expression of TJ proteins (claudin-1, occludin, and THP1) triggered by MW01 treatment. The dynamics of TJ proteins correlates with the gut barrier, as TJ proteins contribute to sealing the paracellular transport between IECs. It has been reported that some probiotic strains confer beneficial effects by immunomodulation and promotion of gut barrier integrity, some of which have been used as a therapeutic or prophylactic approach in humans (Sheil et al., 2007; Arthur et al., 2013). Our findings suggested that *W. cibaria* MW01 may also be used as a potential probiotic reducing the intestinal injury.

The NF-κB-mediated MLCK-pMLC pathway contributes to the modulation of the integrity of the epithelial barrier. NF-κB mediates the upregulation of MLCK, and subsequently, MLCK promotes the phosphorylation of MLC. Then, pMLC disrupts barrier integrity through disassembling the TJ proteins (Gu et al., 2016; Chen et al., 2019). It was noted that *W. cibaria* MW01 reduced the LPS-dependent translocation of NF-κB into the nucleus, indicating the downregulation of the NF-κB signaling pathway. Consistent with these alterations, the upregulation of MCLK and pMLC induced by LPS was also reduced by *W. cibaria* MW01. Considering NF-κB as an important regulatory factor for the expression of pro-inflammatory cytokines, the limitation of the NF-κB pathway by *W. cibaria* MW01 also gained insights into the anti-inflammation mechanism in the present study.

## Conclusion

In conclusion, this study demonstrated the protective effect of *W. cibaria* MW01, derived from Chinese sauerkraut, on the LPS-challenged Caco-2 cell monolayer. MW01 strain adherence to Caco-2 cells effectively attenuated LPS-induced epithelial barrier dysfunction by reducing the pro-inflammatory cytokines such as TNF-α, IL-6, and IL-8. It also inhibited the LPS-induced downregulation of tight-junction proteins claudin-1, occludin, and TJP1 through a mechanism that reduced the nuclear NF-κB translocation and, consequently, the MLCK-pMLC pathways. Even though the further verification of the protective effect of *W. cibaria* MW01 *in vivo* will be more convincible, these results primarily established that *W. cibaria* can regulate intestinal epithelial barrier integrity. Taken together, *W. cibaria* MW01 might be considered as a functional probiotic candidate in the pharmaceutical and food applications.

## Data Availability Statement

All datasets presented in this study are included in the article/Supplementary Material.

## Author Contributions

HZ and WaM designed the experimental strategy. LH and WeM performed the experiments. NY and YD analyzed the experimental data. KC, HZ, and ZL wrote the manuscript. All authors revised and approved the final manuscript.

## Conflict of Interest

The authors declare that the research was conducted in the absence of any commercial or financial relationships that could be construed as a potential conflict of interest.
