# Supplementary material for: Weissella cibaria Attenuated LPS-Induced Dysfunction of Intestinal Epithelial Barrier in a Caco-2 Cell Monolayer Model
Source: Front Microbiol. 2020 Sep 3;11:2039. doi: 10.3389/fmicb.2020.02039 (PMC7509449; doi:10.3389/fmicb.2020.02039)
Supplement: Supplementary file 1 [file Data_Sheet_1.PDF]

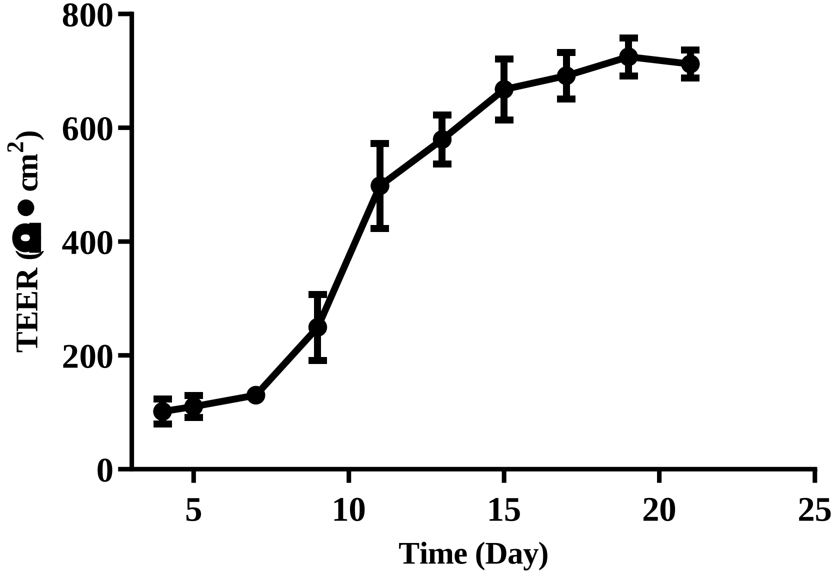

Figure S1. Changes of TEER values at different growth stages.

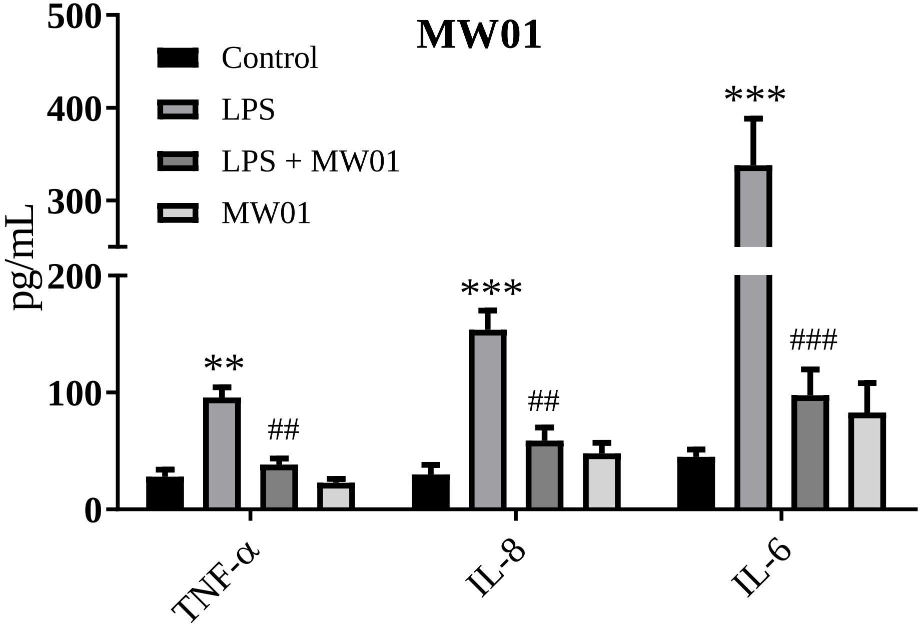

Fig S2 The effect of *W. cibaria* MW01 (A) on secretion of tight pro-inflammatory cytokines in Caco-2 cells challenged with LPS. (**) p<0.005, and (***) p<0.001 versus with control group. (##) p<0.005, and (###) p<0.005 versus with LPS group.
