# Supplementary figures and images for: Weissella cibaria Attenuated LPS-Induced Dysfunction of Intestinal Epithelial Barrier in a Caco-2 Cell Monolayer Model
Source: Front Microbiol. 2020 Sep 3;11:2039. doi: 10.3389/fmicb.2020.02039 (PMC7509449; doi:10.3389/fmicb.2020.02039)

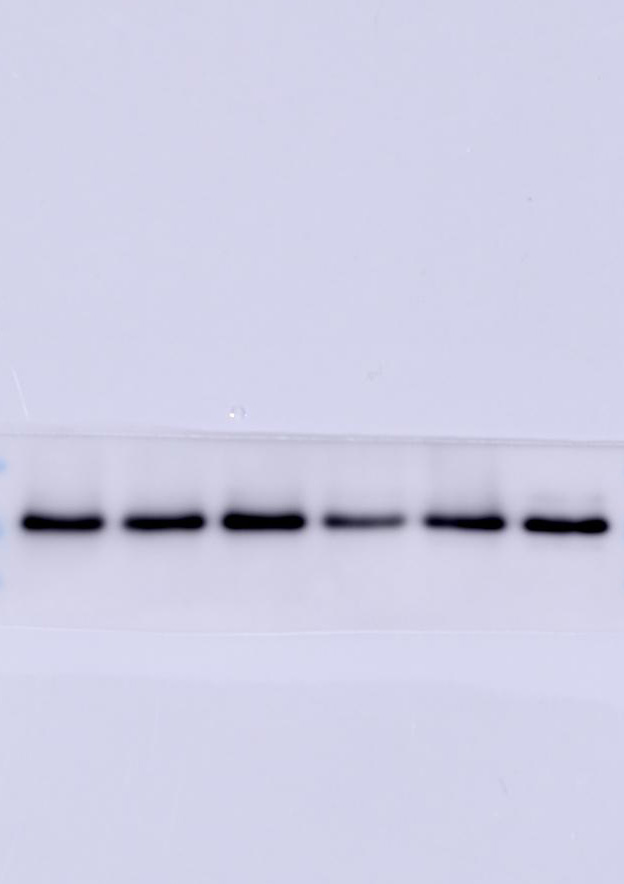

Supplement: Supplementary file 2 [file Data_Sheet_2.zip › Fig5 Claudin-1.jpg]

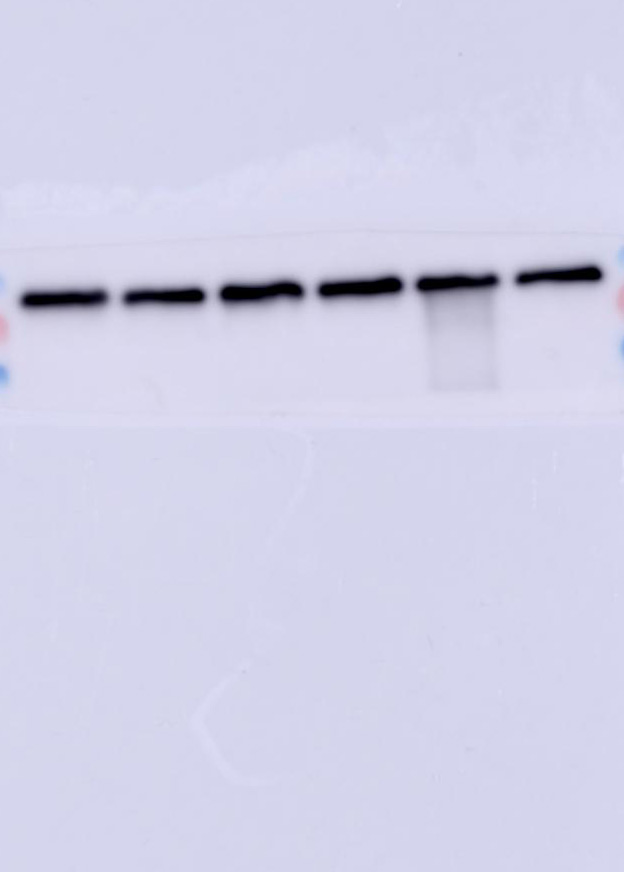

Supplement: Supplementary file 2 [file Data_Sheet_2.zip › Fig5 loading control for Fig 5A and 5B.jpg]

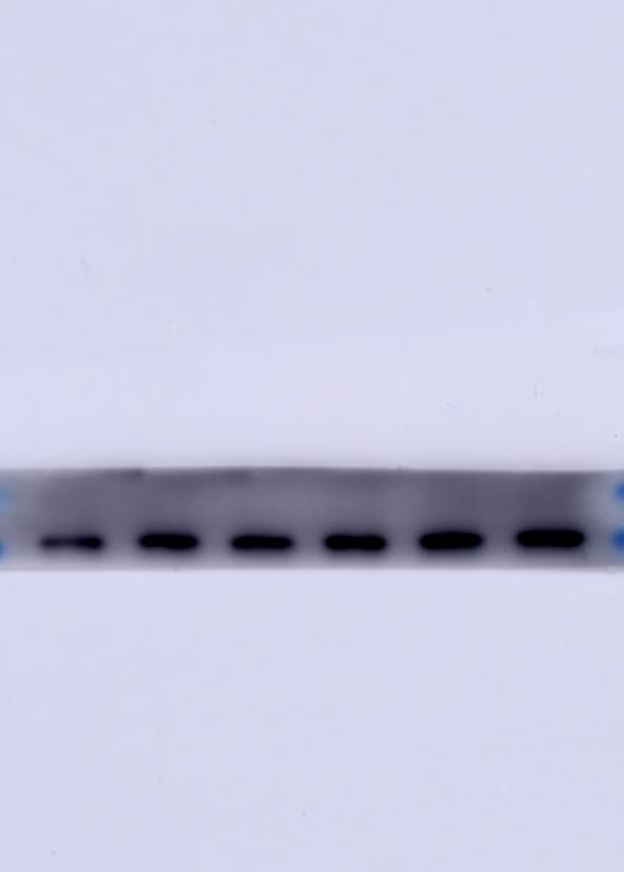

Supplement: Supplementary file 2 [file Data_Sheet_2.zip › Fig5 loading control for Fig 5C.jpg]

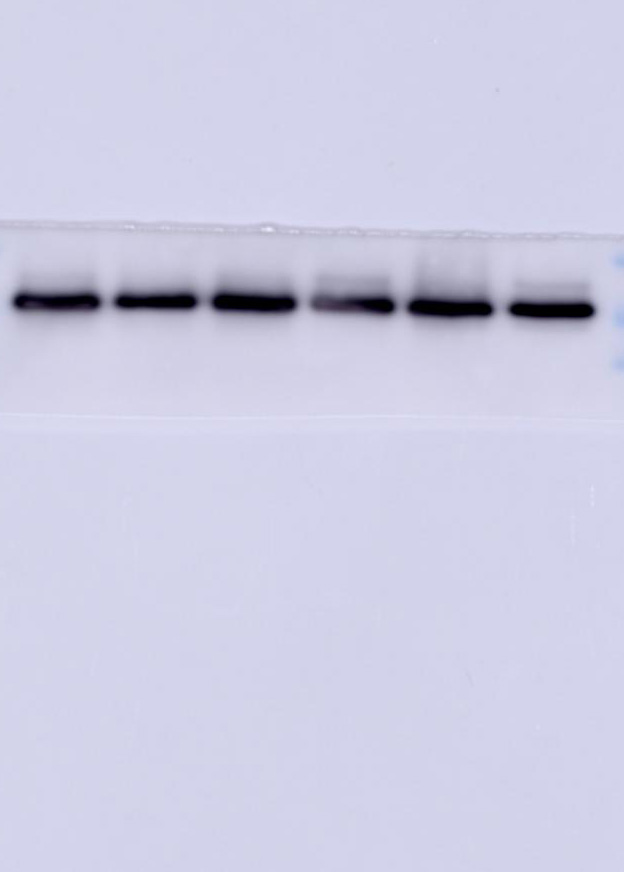

Supplement: Supplementary file 2 [file Data_Sheet_2.zip › Fig5 Occludin.jpg]

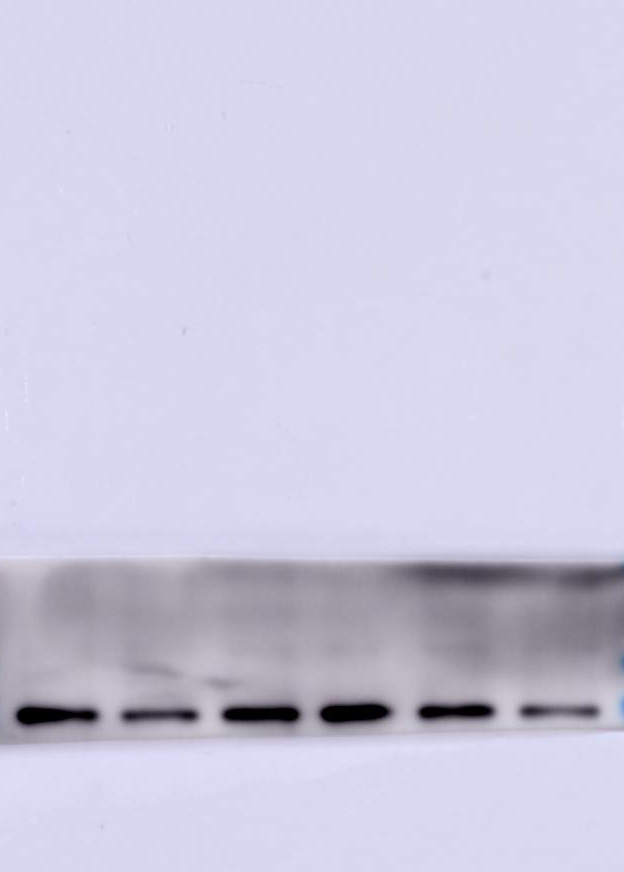

Supplement: Supplementary file 2 [file Data_Sheet_2.zip › Fig5 tight junction protein-1.jpg]

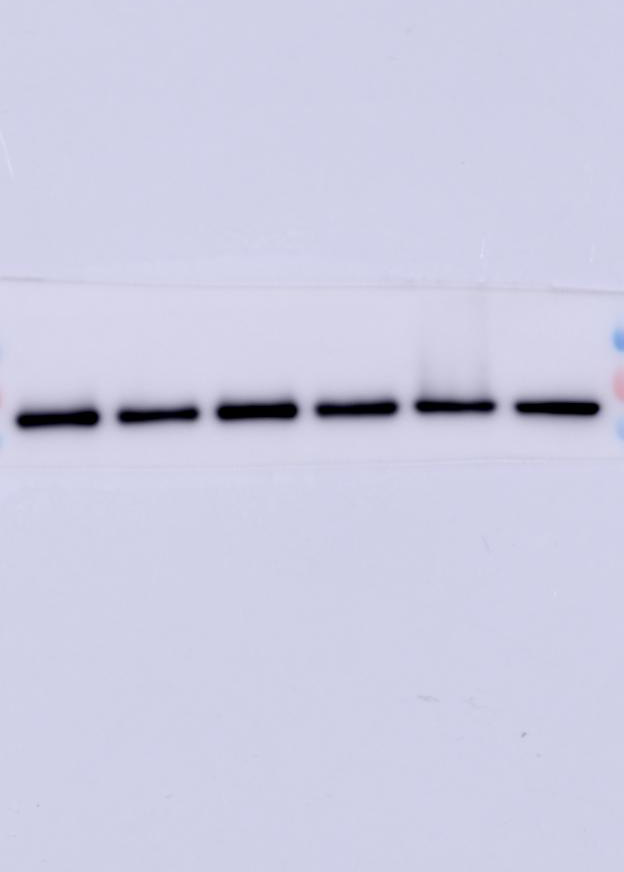

Supplement: Supplementary file 2 [file Data_Sheet_2.zip › Fig6 loading control for MLCK, pMLC and MLC.jpg]

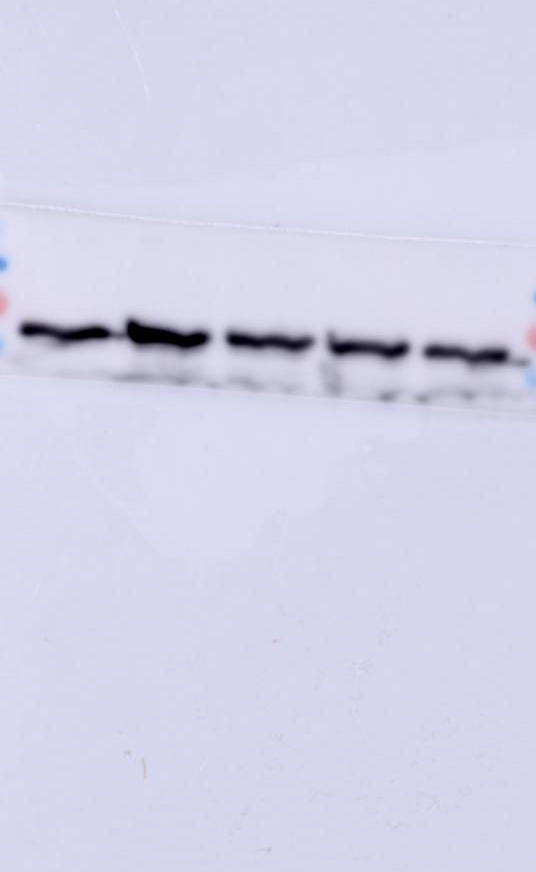

Supplement: Supplementary file 2 [file Data_Sheet_2.zip › Fig6 MLC.jpg]

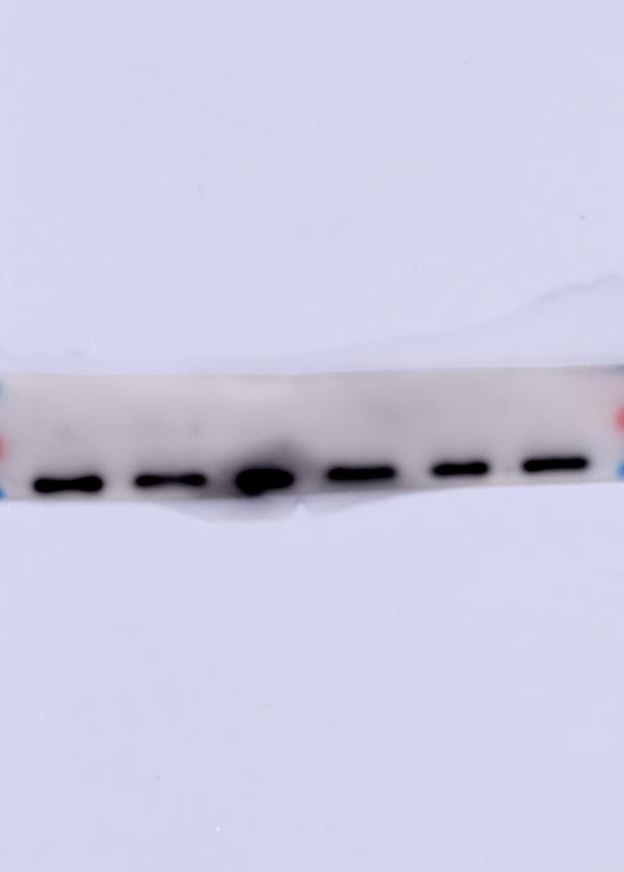

Supplement: Supplementary file 2 [file Data_Sheet_2.zip › Fig6 MLCK.jpg]

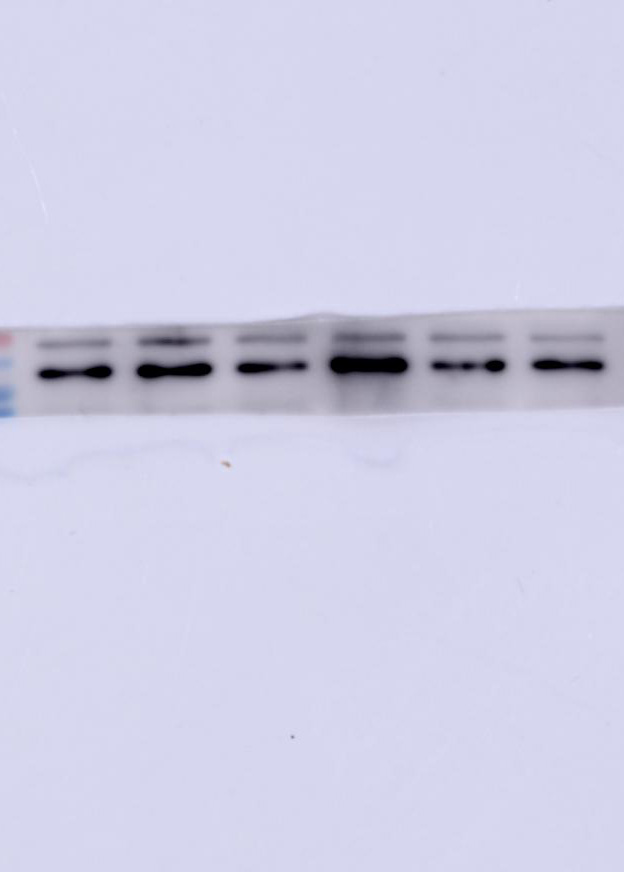

Supplement: Supplementary file 2 [file Data_Sheet_2.zip › Fig6 NF-κB.jpg]

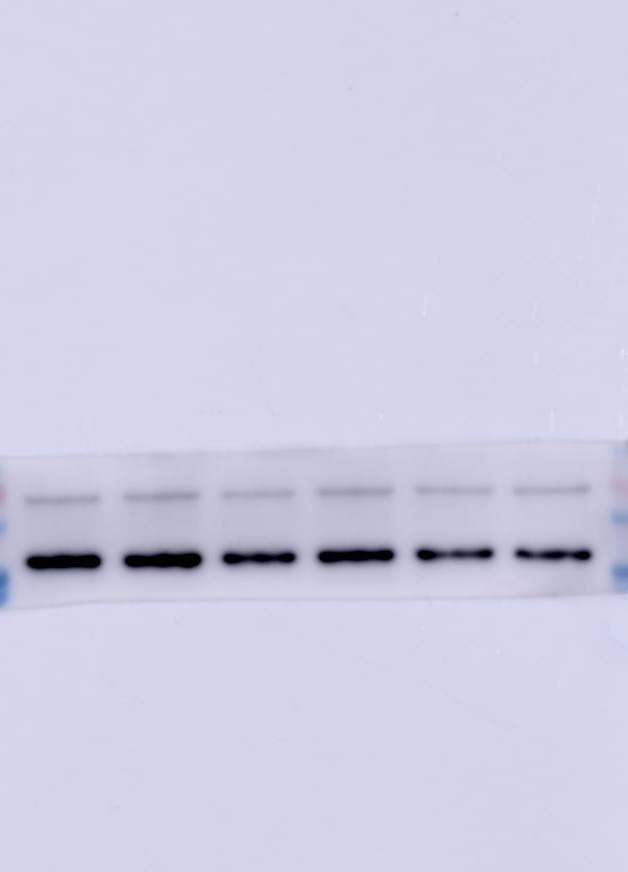

Supplement: Supplementary file 2 [file Data_Sheet_2.zip › Fig6 pMLC.jpg]

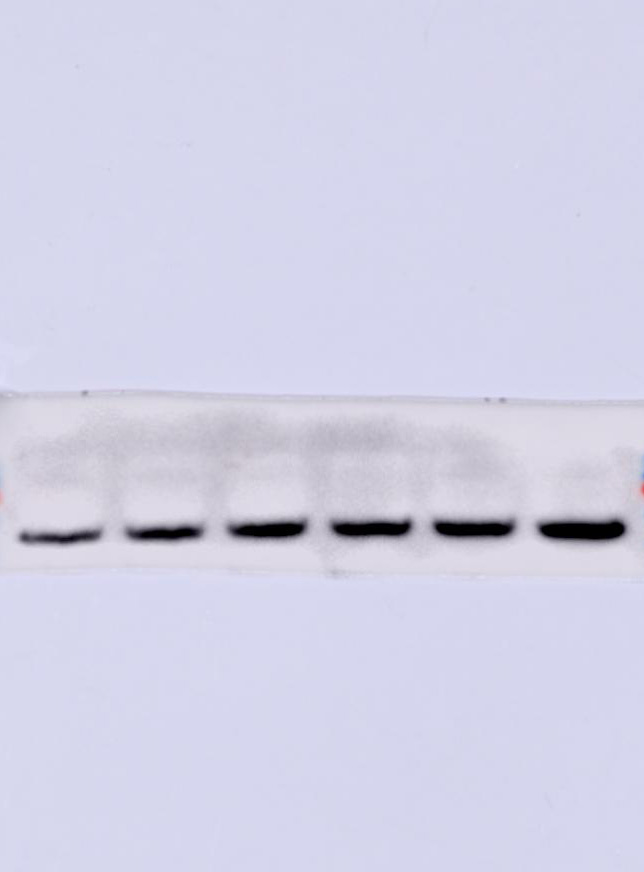

Supplement: Supplementary file 2 [file Data_Sheet_2.zip › Fig6 TBP loading control for NF-κB.jpg]
